# Suppression of Secondary Electron Emission by Vertical Graphene Coating on Ni Microcavity Substrate

**DOI:** 10.3390/nano14151268

**Published:** 2024-07-29

**Authors:** Xiaoning Zhang, Bin Tang, Jialong He, Hui Zhao, Ronghua Wang, Hao Gui, Xinlu Li, Kefu Liu, Jinshui Shi, Guomei Chang

**Affiliations:** 1 School of Information Science and Technology, Fudan University, Shanghai 200433, China; 15681192266@163.com (X.Z.); hui_zhao@fudan.edu.cn (H.Z.); 2 Institute of Fluid Physics (IFP), China Academy of Engineering Physics (CAEP), Mianyang 621900, China; sjs8235@163.com; 3 School of Materials Science and Engineering, Chongqing University, Chongqing 400030, China; 2324644668@163.com (B.T.); wangrh@cqu.edu.cn (R.W.); 15213582609@163.com (H.G.); lixinlu@cqu.edu.cn (X.L.); 4 CSSC-Wuxi Silent Electric System (SES) Technology Co., Ltd., Plot 83-D, National High Tech Development Zone, New District, Xinwu District, No. 1, Xikun Road, Wuxi 214000, China; changguomei@cssc-ses.com

**Keywords:** secondary electron emission, laser etching, microcavity array, vertical graphene, resorption effect

## Abstract

Suppression of secondary electron emission (SEE) from metal surfaces is crucial for enhancing the performance of particle accelerators, spacecraft, and vacuum electronic devices. Earlier research has demonstrated that either etching the metal surface to create undulating structures or coating it with materials having low secondary electron yield (SEY) can markedly decrease SEE. However, the effectiveness of growing vertical graphene (VG) on laser-etched metal surfaces in suppressing SEE remains uncertain. This study examined the collective impact of these methods by applying nanoscale arrays of VG coating using plasma-enhanced chemical vapor deposition on Ni substrates, along with the formation of micrometer-sized microcavity array through laser etching. Comparative tests conducted revealed that the SEY of the samples subjected to VG coating on a microcavity array was lower compared to samples with either only a microcavity array or VG coating alone. Additionally, the crystallinity of VG grown on substrates of varying shapes exhibited variations. This study presents a new method for investigating the suppression of SEE on metal surfaces, contributing to the existing body of knowledge in this field.

## 1. Introduction

Secondary electron emission (SEE) and multiplication on the surface of metal materials can cause the “electron cloud effect” in particle accelerators, the “multipactor effect” in radio-frequency (RF) power devices, and the breakdown of discharges in a variety of devices [1,2,3,4,5]. In order to solve the above problems and improve the performance of related devices, effective SE suppression methods on metal surfaces are important research topics that have been explored by researchers. At present, there are two main types of SE suppression techniques for metal surfaces that have been widely used in related fields. One is to etch the metal surface by various methods, so as to form an array of undulating structures on the metal surface, with a scale ranging from nanometers to millimeters, in order to reduce the secondary electron yield (SEY) of the metal surface through the reabsorption effect of the emitted true SEs from the metal surface by the array of undulating structures. The developed methods of surface etching include mechanical etching, chemical etching, plasma etching, and laser etching, among others [6,7]. The second is to reduce the SEY of metal materials by coating low SEY materials on the metal surface; the developed low SEY coating materials include Ti-Zr-V alloy, TiN, HOPG, amorphous carbon, graphene, etc. [8,9,10,11,12].

It is reported in [13] that the maximum SEY on the sample decreased from 1.9 to 1.12 after employing laser etching to create a microcavity array structure on a Cu surface. Previous research has also confirmed the SE suppression of the microcavity array by employing a laser-etching technique to fabricate a micrometer-scale microcavity array on the metal substrate [13]. Regarding the SE suppression mechanism of the array structure and the microcavity array, SEY reduction primarily stems from the reabsorption of most true SEs emitted from the bottom of the microcavities by the cavity walls, while the SEE performance of the flat regions between the microcavities remains unaltered by the influence of the microcavities. It is hypothesized that further suppressing the SEE from the flat region between the microcavities could lead to further decrease in the SEY of the material surface, thereby achieving a more effective SE suppression effect. In another earlier investigation, we discovered that the vertical graphene (VG) coating on a metal substrate using the plasma-enhanced chemical vapor deposition (PECVD) method can significantly decrease the SEY on the metal surface, resulting in a reduction in the maximum SEY value from 2.3 to 0.75 [14]. It is worth exploring whether an additional VG coating on the metal surface, following the preparation of the microcavity array, can significantly reduce the SEY of the arrays and whether it can achieve a superior SE suppression effect compared to the stand-alone method. Through comprehensive characterization and experiments, this paper demonstrates that combining two approaches will break through the suppression effect of SEE, with VG coating applied after laser etching substantially decreasing the maximum SEY (denoted as δ_m_) of a nickel substrate from 2.30 to 0.57. Furthermore, achieving a δ_m_ below 1 is expected to entirely resolve the issue of SE multiplication. It is shown that VG exhibits varying inhibitory effects on SEs depending on the substrate. This study introduces new research avenues and serves as a reference for developing and modulating carbon materials as novel inhibitors of SEs.

## 2. Materials and Methods

### 2.1. Preparation of Sample

**Figure 1:**
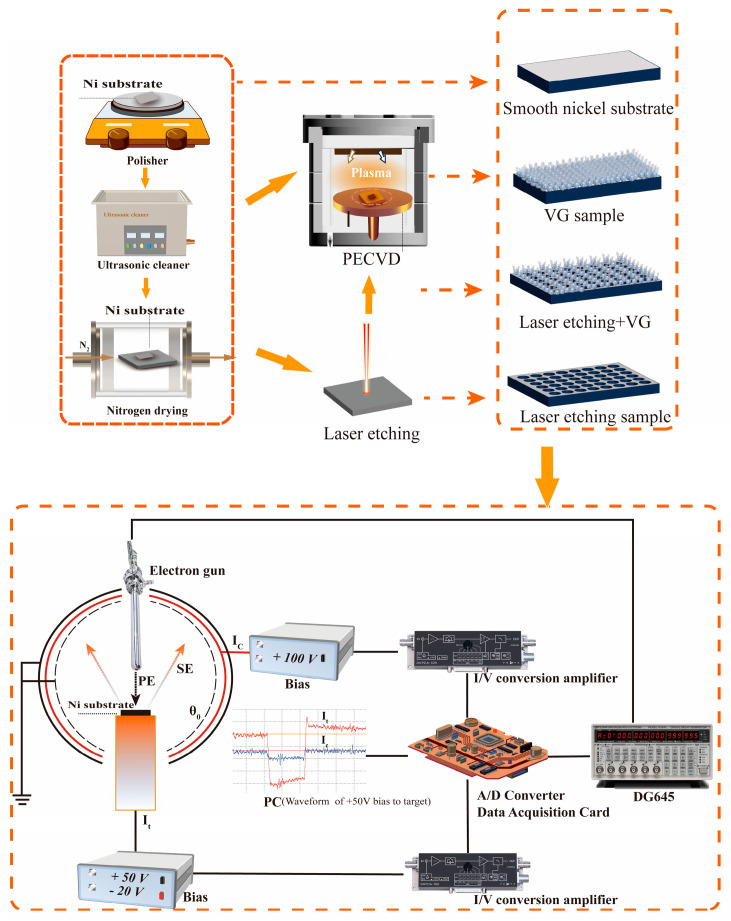
Schematic diagram of the preparation of samples and testing of secondary electron yield.

As shown in Figure 1, we prepared samples featuring a solely laser-etched microcavity array, samples exhibiting only VG coating, and samples with a microcavity array followed by VG coating. The Ni substrate was first roughly ground with 100 grit sandpaper, then finely ground with 500 grit sandpaper until the surface oxidation layer disappeared and presented a metallic luster. Subsequently, the substrate underwent ultrasonic cleaning in deionized water and anhydrous ethanol, followed by nitrogen drying. The substrate surface was etched using an RFL-P pulsed laser. The etching process employed scanning puncturing, with a puncturing etching time of 0.1 ms and a scanning speed of 50 mm/s. The spacing between adjacent holes was 100 μm, with each hole having a diameter of 50 μm and a depth of approximately tens of micrometers. The completed microcavity array preparation samples underwent ultrasonic cleaning with anhydrous ethanol for 30 min, followed by drying and setting aside, and were labeled as LE. Subsequently, VG coating was conducted on both the Ni substrate and the LE samples using PECVD equipment [15]. Initially, the presence of carbon nano-onion coating was observed on the material surfaces. The flow rate ratio of H_2_ to CH_4_ in the device conduit was set at 25:2, while maintaining a pressure of 70 Torr. The plasma power supply was set to 4.2 kW, and the temperature was maintained at 650 °C throughout the 10 min carbon nano-onion growth process and then the sample was cooled to room temperature. Subsequently, VG coating ensued. The flow rate ratio of H_2_ to CH_4_ was adjusted to 10:1, maintaining a pressure of 70 Torr. The power of the plasma power supply remained at 4 kW, and the temperature was controlled at 850 °C throughout the 100 min growth period. The specific parameters are detailed in Table 1. Afterward, the samples were removed following cooling to room temperature. Samples featuring extended VG coating on a smooth nickel substrate and laser-etched substrate were denoted as VG and LE-VG, respectively.

### 2.2. SEE Characteristic Measurement of the Samples

Subsequently, we subjected these samples to SEY testing against the Ni substrate samples. The SE signal emitted from the sample surface was obtained utilizing a spherical electrode-based collection technique, as depicted in the lower half of Figure 1. We conducted experiments to measure the SEY of four types of samples using the collecting pole method. A pulsed electron beam, lasting 10 μs, was emitted from an electron gun and directed through a collimated tube into a spherical SE collector. Each sample was bombarded by an electron beam with a 12 mm diameter spot. The spherical SE collector included a grounded grid to isolate bias voltages between the target and collecting poles. Additionally, a ground grid was installed within the collector for further isolation of bias voltages. The SE energy was regulated by applying +50 V to the target and −20 V to the collector. The incident current and SEs were measured independently to ensure accurate data collection. The application of a +100 V bias voltage to the collector pole prevents SEs received by the collector from re-exciting additional SEs from its surface, thereby reducing the collected SE signal. Detailed experimental methods are available in [16]. The total SE current received by the collector was denoted as I_SE_, the primary electron (PE) current was denoted as $I_{0}$.

According to the definition of SEY, the total SEY, denoted as δ, is

(1) $$\delta =\frac{I_{SE}}{\theta \times I_{0}},$$

the backscattered electron (BE) current received by the collector is denoted as $I_{BE}$, and the backscattered electron yield (BEY), which is denoted as η, can be expressed as

(2) $$\eta =\frac{I_{BE}}{\theta \times I_{0}},$$

Meanwhile, the true SEY, which is denoted as σ, can be expressed as

(3) σ=δ−η

## 3. Results and Discussion

### 3.1. Characterization of VG Coating

**Figure 2:**
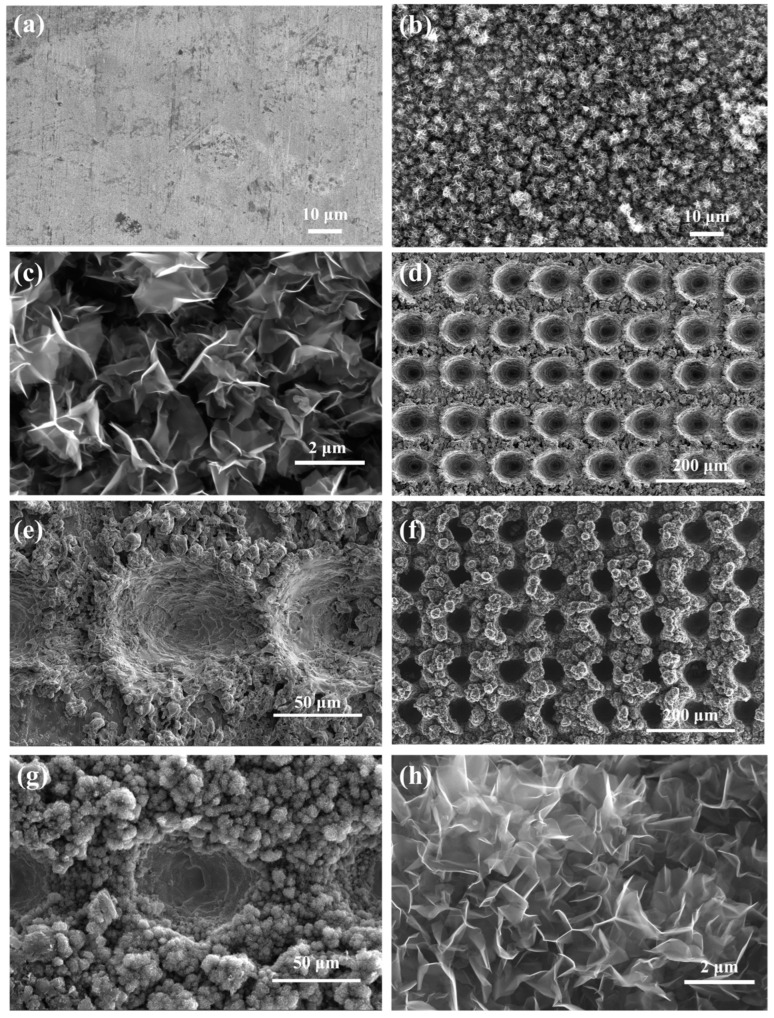
SEM characterization results of the samples. (**a**) Ni substrate, (**b**) VG coating on smooth Ni substrate, (**c**) morphology of VG nanosheets on smooth Ni substrate, (**d**) microcavity array on Ni substrate, (**e**) morphology of microcavities, (**f**) morphology of microcavity array after VG coating, (**g**) distribution of VG in the vicinity of microcavities, and (**h**) the morphology of VG nanosheets at the edges of microcavities.

**Figure 3:**
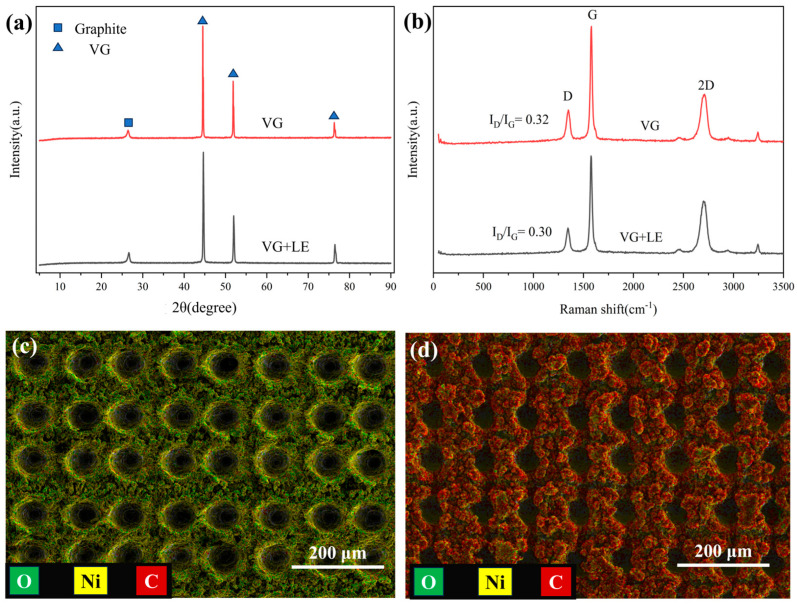
XRD, Raman, and EDS test results of samples. (**a**) XRD comparison of VG on Ni substrate and microcavity array substrate, (**b**) Raman comparison of VG on Ni substrate and microcavity array substrate, (**c**) EDS analysis results of the surface after preparation of microcavity array, (**d**) EDS analysis of the microcavity array after VG coating.

Scanning electron microscope (SEM) test results are depicted in Figure 2, and X-ray diffraction (XRD), Raman, and energy dispersive spectrometer (EDS) test results are displayed in Figure 3. The surface of the unetched and uncoated nickel substrate appears relatively smooth, as shown in Figure 2a. As shown in Figure 2b,c, the VG coating on the smooth Ni substrates covered the entire surface of the sample, and the VG nanosheets were in the range of a few nm in thickness and a few μm in width. Figure 2d,e reveal an array of microcavities formed on the substrate surface after laser etching, with the originally flat areas between the cavities no longer remaining flat due to the accumulation of tiny metal droplets resulting from laser ablation. Figure 2f,g depict VG coating mainly on the substrate surface outside the microcavities of the array structure, with no carbon layer observed on the inner surface of the microcavities. The EDS mapping illustrates a reduction in the oxidized layer on the surface of the microcavity array during VG growth, with the sample surface primarily composed of carbon and a small amount of nickel, as shown in Figure 3c,d. Similarly, the XRD pattern (Figure 3a) shows that the peaks around 2θ = 26° correspond to the characteristic peaks of graphitic carbon, while the peaks around 2θ = 44.5°, 51.8°, and 76.6° can be assigned to the (111), (200), and (220) crystallographic planes of Ni substrates, respectively, which indicates that there is basically no difference in the physical phases after VG coating on the surfaces of the smooth substrates and the microcavity array substrates. Raman spectra of VG are shown in Figure 3b.

There are two characteristic peaks, namely, the D band and G band [17,18], centered at around 1350 cm^−1^ and 1582 cm^−1^, respectively. The appearance of the G band is attributed to the vibration of carbon atoms in sp2 C=C hybridization, which represents the characteristic peak of graphene. The intensity ratio of the D to the G band (I_D_/I_G_) is also employed to characterize defects, with larger values signifying a higher density of defects within graphene. A smaller I_D_/I_G_ value indicates higher graphitization of VG [15], indicating that the graphitization degree and crystallinity of the detected product are high, reflecting the structural characteristics of graphene. This is evident in the comparison between Figure 2c,h, where VG on the microcavity array substrate are more compactly arranged with a greater depth-to-width ratio.

### 3.2. SEY Measurement Results

**Figure 4:**
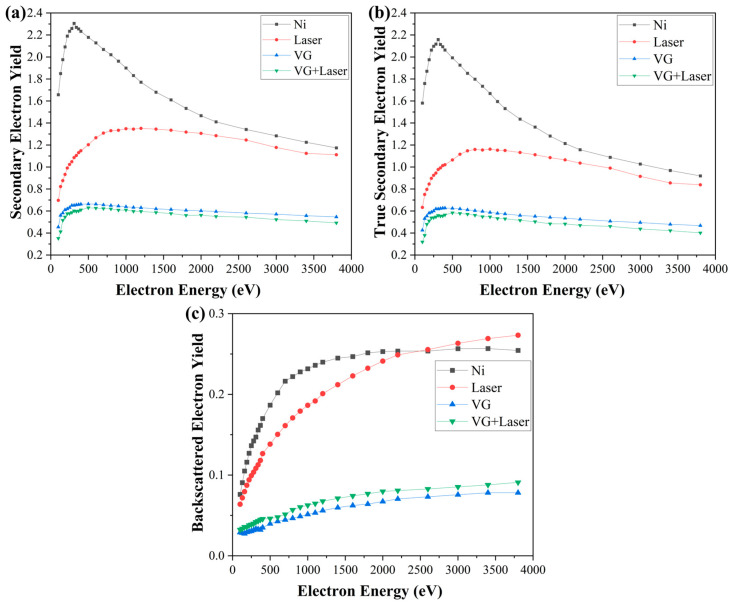
Comparative test results of SEY curves of experimental samples. (**a**) Total SEY curves, (**b**) true SEY curves, (**c**) BEY curves.

The SEY curves of the samples are measured using the collector bias method, as outlined in [19], enabling measurement of the total SEY curve, the true SEY curve, and the BEY curve. The comparative test results of the four samples are illustrated in Figure 4.

The comparative results of the SEY characteristic parameters of the four samples can be derived from the test curves depicted in Figure 4 and demonstrated in Table 2. Comparing LE and LE + VG, the maximum total SEY decreases from 2.30 to 1.34 through laser etching alone. Furthermore, the maximum total SEY decreases further to 0.62 after VG coating, indicating a significant reduction in total SEY. When comparing VG with LE + VG, VG coating alone reduced the maximum total SEY from 2.30 to 0.66. Moreover, the incorporation of a microcavity array on the substrate before VG coating resulted in a lesser additional decrease in SEY. Overall, the coating of VG on a microcavity array substrate leads to further reduction in its SEY. Additionally, the combination of a microcavity array and VG coating produced superior SE suppression compared to a singular method.

### 3.3. SEE Suppression Mechanism

The SEE properties of the material surface are influenced by various factors, such as the surface micromorphology, chemical composition, material morphology, and surface defect distribution [20,21,22,23]. The measurement results indicated a significant decrease in the total SEY after VG coating and laser-etching was applied to the Ni substrate, primarily attributed to the reduction in true SEY. For the surface-etching method, the physical mechanism of SE suppression has been more thoroughly investigated [6,24]. The SEE process on the material surface is influenced by the incident angle of the electrons. When the incident angle is 0°, indicating that the incident electrons are perpendicular to the surface, the travel distance of the incident electrons within the SE escape depth is minimized, leading to reduced excitation and emission of SEs within the material. However, as the incident electron deviates from perpendicular to the surface, increasing the angle of incidence, the range of electron escape depth expands. Consequently, a greater number of SEs can be excited and emitted. SEs in the surface layer of the material exhibit a cosine distribution upon reaching the surface following cascade collisions [25]. When the material surface deviates from a smooth plane to a concave–convex undulating structure, this morphology introduces two effects on the SEE process. On a bumpy undulating surface, the number of SEs emitted increases in regions where the angle of incidence deviates from 0°, as compared to the flat region. Due to the angular distribution of emitted SEs, a significant portion of low-energy true SEs emitted in the concave region collide with the surrounding raised region. Studies have demonstrated that when the material’s surface features an array of undulating structures, the combination of these effects leads to a reduction in emitted SEs compared to a flat surface. Moreover, the extent of this reduction is influenced by the geometrical parameters of the undulating structures, as indicated in prior research [20,26]. Figure 4c illustrates that despite the initial treatment of the Ni substrate with grinding and polishing to eliminate the surface oxide layer, the surface underwent oxidation again during the laser perforation process, resulting in the formation of a new oxide layer. The reduction in the true SEY from 2.1 in the Ni substrate to 1.24 in LE, along with the change of BEY from 0.23 in the substrate to 0.20 in LE, indicates a significant decrease in the true SEY in the Ni substrate due to the reabsorption of true SEs at the bottom by the microcavity array, despite the unfavorable effect of surface oxidation, resulting in a final total SEY reduction from 2.3 to 1.34.

The VG coating on the smooth Ni substrate significantly reduces SEY through two main mechanisms. Firstly, Ref. [27] indicates that carbon materials with an sp2 hybridization mode exhibit better suppression on SEs, leading to reduced SEY after VG coating. Secondly, densely arranged VG nanosheets form an array-type undulating structure similar to a microcavity array, further reducing SEY through reabsorption of the true SEs [28]. This combination leads to a significant reduction in the total SEY value from 2.30 for Ni to 0.66, with the main contribution to the SEY reduction arising from the significant reduction in the true SEY.

Based on the above sample characterization results, it can be inferred that after VG coating on the surface of microcavity array, the VG are mainly distributed outside the microcavities. In comparison to the microcavity array substrate, the oxidized layer on the surface was once more removed during VG coating due to the reducing atmosphere. The VG coating between the microcavities, while retaining its own true SE reabsorption effect on the bottom of the microcavities, exerted a similar effect as VG coating on a flat substrate, leading to a notable reduction in the SEY within the flat region between the microcavities. The SEY suppression effect of both the microcavities themselves and the VG overlay between them resulted in LE + VG exhibiting a superior SE suppression effect compared to the individual methods. The small SEY reduction in LE + VG compared to VG alone suggests a considerable SE suppression effect of VGs. Additionally, the further SEY suppression effect resulting from the introduction of additional micrometer-scale array structures on a flat substrate is relatively limited for VG coating.

## 4. Conclusions

In this study, the utilization of laser-etching and VG coating led to a substantial 42% reduction to 0.62 in SEY, addressing the effects of SE multiplication. Furthermore, the subsequent application of VG coating on the microcavity array substrate using plasma-enhanced chemical vapor deposition resulted in an impressive 73% reduction in SEY, slightly surpassing the 71% reduction achieved with VG coating alone. These results clearly demonstrate that the combination of surface etching and VG coating techniques can yield superior suppression of SEE compared to employing these methods individually. In addition, this study reveals that the crystallinity of VG varies on substrates of different shapes, and the VG nanosheets grown on the surface of the microcavity array substrate are more densely arranged with a higher depth-to-width ratio. These findings provide valuable and novel insights into nanocarbon applications in the field of metallic SE suppression.

## Figures and Tables

**Table 1:** The key parameters and values of PECVD for preparing VG coating with well on the substrates.

Parameters	Flow Rate of H_2_ and CH_4_	Pressure (Torr)	Plasma Power (kW)	Temperature (°C)	Time (min)
Value	25:2	70	4.2	650	30
Value	10:1	70	4	850	100

**Table 2:** Comparison of SEE characteristic parameters of four samples.

Sample	δ_max_	δ_max_ Decreases Relative to Ni	σ_max_	σ_max_ Decreases Relative to Ni	BEY with Maximum δ_max_
Ni	2.30	-	2.1	-	0.23
LE	1.34	42%	1.24	41%	0.20
VG	0.66	71%	0.62	70%	0.04
LE + VG	0.62	73%	0.57	73%	0.04

## Data Availability

Data is contained within the article.
